# Subjective cognitive impairment in bipolar disorder patients: prevalence and associated factors

**DOI:** 10.1192/j.eurpsy.2025.1082

**Published:** 2025-08-26

**Authors:** F. Sahnoun, F. Cherif, D. Mnif, F. Guermazi, I. Baati, I. Feki, R. Masmoudi, J. Masmoudi

**Affiliations:** 1 Psychiatry A Department, Hedi Chaker University hospital, Sfax, Tunisia

## Abstract

**Introduction:**

Over the last decade, there has been a growing recognition of the importance of identifying and treating cognitive impairment associated with bipolar disorder, as it persists during remission periods. Evidence suggests that neurocognitive dysfunction may significantly influence patients’ psychosocial outcomes. An increasing body of research aims to improve understanding of potential moderators contributing to cognitive impairment in bipolar disorder in order to develop prevention strategies and effective treatments

**Objectives:**

The aim of this study is to explore the prevalence of cognitive impairment among bipolar disorder patients and identify related factors.

**Methods:**

It was a cross-sectional, descriptive, and analytical study conducted on bipolar disorder patients from the Psychiatry “A” Department, Hedi Chaker University Hospital. Clinical and sociodemographic data were collected from March to September 2023 through a questionnaire along with The Cognitive Complaints in Bipolar Disorder Rating Assessment (COBRA) for evaluation of subjective cognitive impairment

**Results:**

A total of 37 patients with BD completed the questionnaire. The mean age was 45.4 ± 13.9 years, with a sex ratio (M/F) of 1.46.

Our results showed that 73 % of patients were with BD type I and 27% were with BD type II.

The mean score of COBRA was 12.54 ± 7.62 and 32.4% of participants presented subjective cognitive disorder.

Subjective cognitive disorder was significantly associated with the number of relapses, hospitalizations and suicide attempts, with respectively p< 0.001, p= 0.02 and p= 0.05

Female patients and patients with poor income presented significantly more subjective cognitive disorders (p = 0.01 and p = 0.02, respectively).

The COBRA score was positively correlated with the number of relapses (p<0.001, r = 0.67).

**Conclusions:**

Our findings indicate that a significant proportion of individuals with bipolar disorder report cognitive difficulties, which may impact their daily functioning and quality of life. Key factors such as poor income, female sex, and the number of relapses and suicide attempts were associated with higher levels of perceived cognitive dysfunction. These results highlight the need for greater attention to cognitive symptoms in the clinical management of bipolar disorder.

**Disclosure of Interest:**

None Declared

